# 4-Chloro-*N*-(2-methoxy­phen­yl)benzamide

**DOI:** 10.1107/S160053680905394X

**Published:** 2009-12-19

**Authors:** Aamer Saeed, Rasheed Ahmad Khera, Jim Simpson

**Affiliations:** a Department of Chemistry, Quaid-i-Azam University, Islamabad 45320, Pakistan; b Department of Chemistry, University of Otago, PO Box 56, Dunedin, New Zealand

## Abstract

The title compound, C_14_H_12_ClNO_2_, was prepared by refluxing 4-chloro­benzoyl chloride with *o*-anisidine in CHCl_3_. The methoxy­phen­yl–amide segment of the mol­ecule is almost planar, with a dihedral angle of 5.10 (7)° between the benzene ring and the C—N—C(O)—C fragment. A weak intra­molecular N—H⋯O contact forms an *S*(5) ring and contributes to the planarity of this portion of the mol­ecule. The two benzene rings are inclined at an angle of 26.74 (7)°. In the crystal structure, inter­molecular Cl⋯O inter­actions of 3.1874 (9) Å generate centrosymmetric dimers. These are further linked by C—H⋯O and C—H⋯π inter­actions, forming inversion related sheets parallel to [001].

## Related literature

For background to our work on benzamide derivatives, see: Saeed *et al.* (2008 ▶). For related structures, see: Balasubramanyam *et al.* (2003 ▶); Gowda *et al.* (2008 ▶); Saeed *et al.* (2007 ▶). For hydrogen-bond motifs, see: Bernstein *et al.* (1995 ▶).
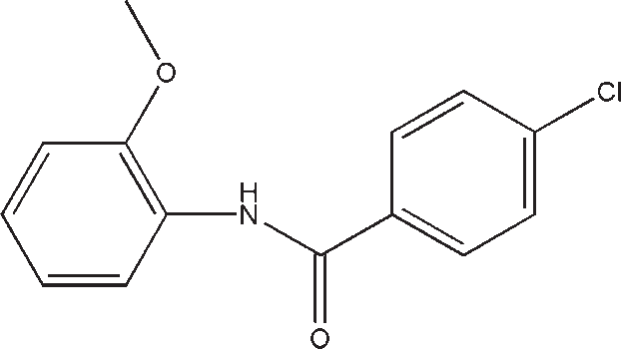


## Experimental

### 

#### Crystal data

C_14_H_12_ClNO_2_
*M* *_r_* = 261.70
Triclinic, 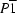

*a* = 7.6938 (5) Å
*b* = 9.2339 (6) Å
*c* = 9.8723 (7) Å
α = 66.683 (3)°
β = 89.943 (3)°
γ = 69.536 (3)°
*V* = 595.69 (7) Å^3^
*Z* = 2
Mo *K*α radiation
μ = 0.31 mm^−1^
*T* = 89 K
0.68 × 0.55 × 0.38 mm

#### Data collection

Bruker APEXII CCD area-detector diffractometer
Absorption correction: multi-scan (*SADABS*; Bruker, 2006 ▶) *T*_min_ = 0.762, *T*_max_ = 1.000
9701 measured reflections
4037 independent reflections
3359 reflections with *I* > 2σ(*I*)
*R*_int_ = 0.034

#### Refinement

*R*[*F* ^2^ > 2σ(*F* ^2^)] = 0.039
*wR*(*F* ^2^) = 0.129
*S* = 1.11
4037 reflections
167 parameters
H atoms treated by a mixture of independent and constrained refinement
Δρ_max_ = 0.49 e Å^−3^
Δρ_min_ = −0.41 e Å^−3^

### 

Data collection: *APEX2* (Bruker, 2006 ▶); cell refinement: *APEX2* and *SAINT* (Bruker, 2006 ▶); data reduction: *SAINT*; program(s) used to solve structure: *SHELXS97* (Sheldrick, 2008 ▶) and *TITAN2000* (Hunter & Simpson, 1999 ▶); program(s) used to refine structure: *SHELXL97* (Sheldrick, 2008 ▶) and *TITAN2000*; molecular graphics: *SHELXTL* (Sheldrick, 2008 ▶) and *Mercury* (Macrae *et al.*, 2006 ▶); software used to prepare material for publication: *SHELXL97*, *enCIFer* (Allen *et al.*, 2004 ▶), *PLATON* (Spek, 2009 ▶) and *publCIF* (Westrip, 2010 ▶).

## Supplementary Material

Crystal structure: contains datablocks global, I. DOI: 10.1107/S160053680905394X/bq2183sup1.cif

Structure factors: contains datablocks I. DOI: 10.1107/S160053680905394X/bq2183Isup2.hkl

Additional supplementary materials:  crystallographic information; 3D view; checkCIF report

## Figures and Tables

**Table 1:** Hydrogen-bond geometry (Å, °) *Cg*1 is the centroid of the C8–C13 ring.

*D*—H⋯*A*	*D*—H	H⋯*A*	*D*⋯*A*	*D*—H⋯*A*
N1—H1*N*⋯O91	0.855 (17)	2.165 (19)	2.5810 (16)	109.7 (15)
C4—H4⋯O1^i^	0.95	2.37	3.3060 (15)	167
C6—H6⋯*Cg*1^ii^	0.95	3.33	3.911 (2)	133

Symmetry codes: (i) 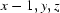; (ii) 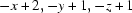.

**Fig. 1.**
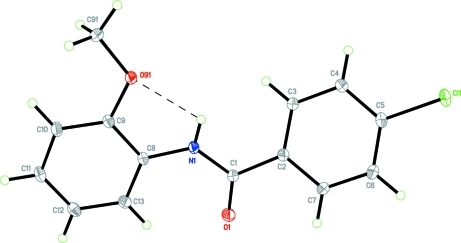
The structure of (I) with displacement ellipsoids for the non-hydrogen atoms drawn at the 50% probability level. The intramolecular hydrogen bond is drawn as a dashed line.

**Fig. 2.**
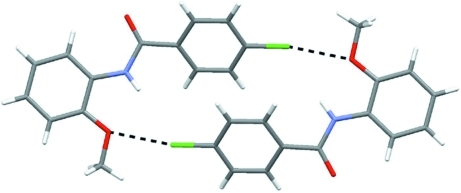
Cl···O contacts in (I) (dashed lines) linking the molecules into centrosymmetric dimers.

**Fig. 3.**
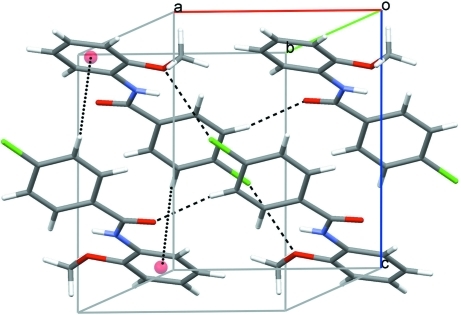
Crystal packing of (I) viewed down the b axis, with hydrogen bonds drawn as dashed lines and representative C—H···π interactions shown as dotted lines. Red spheres represent the centroids of the C8···C13 rings.

## supplementary crystallographic information

### Comment

Our work on benzamide derivatives has been described in a previous paper (Saeed *et al.*, 2008). The methoxyphenyl amide segment of the molecule is planar with a dihedral angle of 5.10 (7) ° between benzene ring and the C8—N1—C1(O1)—C2 fragment. A weak intramolecular N1—H1N···O91 contact forms an S(5) ring (Bernstein *et al.*, 1995) and contributes to the planarity of this portion of the molecule. The O91 and C91 atoms of the methoxy group also lie close to the C8···C13 ring plane with deviations 0.0171 (17) Å for O91 and -0.040 (2)Å for C91 respectively. The two benzene rings are inclined at an angle of 26.74 (7) °. Bond distances within the molecule are similar to those observed in comparable structures (Balasubramanyam *et al.*,2003; Saeed *et al.*, 2007; Gowda *et al.*, 2008).

In the crystal structure intermolecular Cl1···O1 interactions, 3.1874 (9) Å, generate centrosymmetric dimers, Fig. 2. Molecules in these dimers are further linked by C4—H4···O1 and C6—H6···*Cg* interactions (*Cg* is the centroid of the C8···C13 ring), Table 1, forming inversion related sheets parallel to 001, Fig 3.

### Experimental

A freshly prepared solution of 4-chlorobenzoyl chloride (1 mmol) in CHCl_3_ was treated with *o*-anisidine (1 mmol) under a nitrogen atmosphere at reflux for 2.5 h. Upon cooling, the reaction mixture was diluted with CHCl_3_ and washed consecutively with 1 *M* aq HCl and saturated aq NaHCO_3_. The organic layer was dried over anhydrous sodium sulfate and concentrated under reduced pressure. Crystallization of the residue from methanol afforded the title compound (87%) as colourless crystals: Anal. calcd. for C_14_H_12_ClNO2: C, 64.25; H, 4.62; N, 5.35%; found: C, 64.09; H, 4.71; N, 5.43%.

### Refinement

The H atom on N1 was located in a difference Fourier map and refined isotropically. All other H-atoms were placed in calculated positions and refined using a riding model with d(C—H) = 0.95 Å, *U*_iso_ = 1.2*U*_eq_ (C) for aromatic and 0.98 Å, *U*_iso_ = 1.5*U*_eq_ (C) for the CH_3_ H atoms. The crystal was relatively weakly diffracting reducing the overall fraction of measured reflections.

### Crystal data

**Table:**

C_14_H_12_ClNO_2_	*Z* = 2
*M**_r_* = 261.70	*F*(000) = 272
Triclinic, *P*1	*D*_x_ = 1.459 Mg m^−^^3^
Hall symbol: -P 1	Mo *K*α radiation, λ = 0.71073 Å
*a* = 7.6938 (5) Å	Cell parameters from 5193 reflections
*b* = 9.2339 (6) Å	θ = 5.2–66.5°
*c* = 9.8723 (7) Å	µ = 0.31 mm^−^^1^
α = 66.683 (3)°	*T* = 89 K
β = 89.943 (3)°	Irregular block, colourless
γ = 69.536 (3)°	0.68 × 0.55 × 0.38 mm
*V* = 595.69 (7) Å^3^	

### Data collection

**Table:**

Bruker APEXII CCD area-detector diffractometer	4037 independent reflections
Radiation source: fine-focus sealed tube	3359 reflections with *I* > 2σ(*I*)
graphite	*R*_int_ = 0.034
ω scans	θ_max_ = 33.4°, θ_min_ = 3.4°
Absorption correction: multi-scan (*SADABS*; Bruker, 2006)	*h* = −10→11
*T*_min_ = 0.762, *T*_max_ = 1.000	*k* = −14→14
9701 measured reflections	*l* = −15→14

### Refinement

**Table:**

Refinement on *F*^2^	Primary atom site location: structure-invariant direct methods
Least-squares matrix: full	Secondary atom site location: difference Fourier map
*R*[*F*^2^ > 2σ(*F*^2^)] = 0.039	Hydrogen site location: inferred from neighbouring sites
*wR*(*F*^2^) = 0.129	H atoms treated by a mixture of independent and constrained refinement
*S* = 1.11	*w* = 1/[σ^2^(*F*_o_^2^) + (0.0747*P*)^2^ + 0.107*P*] where *P* = (*F*_o_^2^ + 2*F*_c_^2^)/3
4037 reflections	(Δ/σ)_max_ = 0.001
167 parameters	Δρ_max_ = 0.49 e Å^−^^3^
0 restraints	Δρ_min_ = −0.41 e Å^−^^3^

### Special details

**Table:**

Geometry. All e.s.d.'s (except the e.s.d. in the dihedral angle between two l.s. planes) are estimated using the full covariance matrix. The cell e.s.d.'s are taken into account individually in the estimation of e.s.d.'s in distances, angles and torsion angles; correlations between e.s.d.'s in cell parameters are only used when they are defined by crystal symmetry. An approximate (isotropic) treatment of cell e.s.d.'s is used for estimating e.s.d.'s involving l.s. planes.
Refinement. Refinement of *F*^2^ against ALL reflections. The weighted *R*-factor *wR* and goodness of fit *S* are based on *F*^2^, conventional *R*-factors *R* are based on *F*, with *F* set to zero for negative *F*^2^. The threshold expression of *F*^2^ > σ(*F*^2^) is used only for calculating *R*-factors(gt) *etc*. and is not relevant to the choice of reflections for refinement. *R*-factors based on *F*^2^ are statistically about twice as large as those based on *F*, and *R*- factors based on ALL data will be even larger.

### Fractional atomic coordinates and isotropic or equivalent isotropic displacement parameters (Å^2^)

**Table:**

	*x*	*y*	*z*	*U*_iso_*/*U*_eq_	
N1	0.96801 (14)	0.46684 (13)	0.22602 (12)	0.01253 (19)	
H1N	0.884 (2)	0.424 (2)	0.2472 (19)	0.015*	
O1	1.05224 (13)	0.67559 (12)	0.23889 (11)	0.01810 (19)	
C1	0.94176 (16)	0.60270 (14)	0.25817 (13)	0.0119 (2)	
C2	0.76731 (16)	0.65786 (14)	0.32264 (13)	0.0115 (2)	
C3	0.60538 (16)	0.63062 (15)	0.29623 (13)	0.0128 (2)	
H3	0.6034	0.5737	0.2348	0.015*	
C4	0.44720 (16)	0.68559 (15)	0.35862 (14)	0.0137 (2)	
H4	0.3370	0.6679	0.3396	0.016*	
C5	0.45387 (16)	0.76691 (15)	0.44926 (13)	0.0134 (2)	
Cl1	0.25804 (4)	0.83363 (4)	0.53040 (3)	0.01918 (10)	
C6	0.61287 (17)	0.79600 (15)	0.47773 (13)	0.0141 (2)	
H6	0.6148	0.8514	0.5405	0.017*	
C7	0.76861 (16)	0.74244 (15)	0.41260 (13)	0.0129 (2)	
H7	0.8772	0.7635	0.4294	0.015*	
C8	1.12198 (15)	0.38098 (14)	0.17333 (13)	0.0111 (2)	
C9	1.11216 (15)	0.24043 (14)	0.15384 (13)	0.0119 (2)	
O91	0.95021 (12)	0.21277 (11)	0.18506 (10)	0.01454 (18)	
C91	0.92918 (17)	0.07592 (16)	0.16205 (15)	0.0170 (2)	
H91A	0.9212	0.1017	0.0554	0.026*	
H91B	0.8142	0.0620	0.1967	0.026*	
H91C	1.0378	−0.0297	0.2183	0.026*	
C10	1.25940 (16)	0.14161 (15)	0.10850 (14)	0.0142 (2)	
H10	1.2514	0.0479	0.0946	0.017*	
C11	1.41951 (17)	0.18041 (16)	0.08331 (14)	0.0156 (2)	
H11	1.5219	0.1115	0.0541	0.019*	
C12	1.42992 (17)	0.31922 (16)	0.10069 (14)	0.0156 (2)	
H12	1.5391	0.3453	0.0826	0.019*	
C13	1.28124 (16)	0.42081 (15)	0.14455 (14)	0.0139 (2)	
H13	1.2883	0.5167	0.1548	0.017*	

### Atomic displacement parameters (Å^2^)

**Table:**

	*U*^11^	*U*^22^	*U*^33^	*U*^12^	*U*^13^	*U*^23^
N1	0.0111 (4)	0.0137 (4)	0.0177 (5)	−0.0062 (3)	0.0069 (4)	−0.0100 (4)
O1	0.0149 (4)	0.0190 (4)	0.0284 (5)	−0.0099 (3)	0.0084 (4)	−0.0148 (4)
C1	0.0107 (5)	0.0131 (5)	0.0137 (5)	−0.0044 (4)	0.0027 (4)	−0.0073 (4)
C2	0.0111 (5)	0.0111 (4)	0.0125 (5)	−0.0037 (4)	0.0023 (4)	−0.0057 (4)
C3	0.0118 (5)	0.0143 (5)	0.0147 (5)	−0.0051 (4)	0.0034 (4)	−0.0084 (4)
C4	0.0115 (5)	0.0152 (5)	0.0160 (5)	−0.0054 (4)	0.0037 (4)	−0.0078 (4)
C5	0.0132 (5)	0.0130 (5)	0.0134 (5)	−0.0035 (4)	0.0047 (4)	−0.0062 (4)
Cl1	0.01588 (16)	0.02343 (17)	0.02268 (17)	−0.00678 (12)	0.00998 (12)	−0.01468 (13)
C6	0.0162 (5)	0.0139 (5)	0.0143 (5)	−0.0053 (4)	0.0037 (4)	−0.0083 (4)
C7	0.0129 (5)	0.0133 (5)	0.0145 (5)	−0.0051 (4)	0.0023 (4)	−0.0078 (4)
C8	0.0100 (4)	0.0108 (4)	0.0118 (5)	−0.0029 (4)	0.0029 (4)	−0.0052 (4)
C9	0.0103 (5)	0.0124 (5)	0.0128 (5)	−0.0039 (4)	0.0031 (4)	−0.0056 (4)
O91	0.0123 (4)	0.0148 (4)	0.0228 (5)	−0.0073 (3)	0.0080 (3)	−0.0122 (3)
C91	0.0149 (5)	0.0163 (5)	0.0261 (6)	−0.0077 (4)	0.0052 (5)	−0.0135 (5)
C10	0.0125 (5)	0.0134 (5)	0.0171 (5)	−0.0035 (4)	0.0047 (4)	−0.0082 (4)
C11	0.0113 (5)	0.0168 (5)	0.0178 (6)	−0.0028 (4)	0.0054 (4)	−0.0085 (4)
C12	0.0115 (5)	0.0177 (5)	0.0172 (5)	−0.0059 (4)	0.0048 (4)	−0.0068 (4)
C13	0.0128 (5)	0.0149 (5)	0.0161 (5)	−0.0068 (4)	0.0045 (4)	−0.0074 (4)

### Geometric parameters (Å, °)

**Table:**

N1—C1	1.3613 (14)	C7—H7	0.9500
N1—C8	1.4039 (13)	C8—C13	1.3961 (15)
N1—H1N	0.860 (17)	C8—C9	1.4119 (15)
O1—C1	1.2288 (13)	C9—O91	1.3683 (13)
C1—C2	1.4977 (15)	C9—C10	1.3841 (15)
C2—C7	1.3977 (15)	O91—C91	1.4301 (13)
C2—C3	1.3982 (15)	C91—H91A	0.9800
C3—C4	1.3901 (15)	C91—H91B	0.9800
C3—H3	0.9500	C91—H91C	0.9800
C4—C5	1.3879 (16)	C10—C11	1.3947 (16)
C4—H4	0.9500	C10—H10	0.9500
C5—C6	1.3920 (16)	C11—C12	1.3873 (16)
C5—Cl1	1.7408 (11)	C11—H11	0.9500
Cl1—O91^i^	3.1874 (9)	C12—C13	1.3951 (16)
C6—C7	1.3891 (15)	C12—H12	0.9500
C6—H6	0.9500	C13—H13	0.9500
			
C1—N1—C8	128.16 (9)	C13—C8—C9	119.25 (10)
C1—N1—H1N	115.7 (11)	N1—C8—C9	115.41 (9)
C8—N1—H1N	116.0 (11)	O91—C9—C10	124.96 (10)
O1—C1—N1	123.69 (10)	O91—C9—C8	114.45 (9)
O1—C1—C2	121.14 (10)	C10—C9—C8	120.58 (10)
N1—C1—C2	115.16 (9)	C9—O91—C91	117.00 (9)
C7—C2—C3	119.14 (10)	O91—C91—H91A	109.5
C7—C2—C1	117.28 (10)	O91—C91—H91B	109.5
C3—C2—C1	123.57 (10)	H91A—C91—H91B	109.5
C4—C3—C2	120.95 (10)	O91—C91—H91C	109.5
C4—C3—H3	119.5	H91A—C91—H91C	109.5
C2—C3—H3	119.5	H91B—C91—H91C	109.5
C5—C4—C3	118.53 (10)	C9—C10—C11	119.63 (10)
C5—C4—H4	120.7	C9—C10—H10	120.2
C3—C4—H4	120.7	C11—C10—H10	120.2
C4—C5—C6	121.93 (10)	C12—C11—C10	120.28 (10)
C4—C5—Cl1	119.03 (9)	C12—C11—H11	119.9
C6—C5—Cl1	119.04 (9)	C10—C11—H11	119.9
C7—C6—C5	118.73 (10)	C11—C12—C13	120.49 (10)
C7—C6—H6	120.6	C11—C12—H12	119.8
C5—C6—H6	120.6	C13—C12—H12	119.8
C6—C7—C2	120.71 (11)	C12—C13—C8	119.75 (10)
C6—C7—H7	119.6	C12—C13—H13	120.1
C2—C7—H7	119.6	C8—C13—H13	120.1
C13—C8—N1	125.31 (10)		

Symmetry codes: (i) −*x*+1, −*y*+1, −*z*+1.

### Hydrogen-bond geometry (Å, °)

**Table:**

Cg1 is the centroid of the C8–C13 ring.

**Table:**

*D*—H···*A*	*D*—H	H···*A*	*D*···*A*	*D*—H···*A*
N1—H1N···O91	0.855 (17)	2.165 (19)	2.5810 (16)	109.7 (15)
C4—H4···O1^ii^	0.95	2.37	3.3060 (15)	167
C6—H6···Cg1^iii^	0.95	3.33	3.911 (2)	133

Symmetry codes: (ii) *x*−1, *y*, *z*; (iii) −*x*+2, −*y*+1, −*z*+1.
